# The Role of Neuronal Factors in the Epigenetic Reprogramming of Microglia in the Normal and Diseased Central Nervous System

**DOI:** 10.3389/fncel.2019.00453

**Published:** 2019-10-11

**Authors:** Tatyana Veremeyko, Amanda W. Y. Yung, Marina Dukhinova, Tatyana Strekalova, Eugene D. Ponomarev

**Affiliations:** ^1^School of Biomedical Sciences, Faculty of Medicine, The Chinese University of Hong Kong, Shatin, Hong Kong; ^2^Synthetic and Systems Biology for Biomedicine, Italian Institute of Technology (IIT), Genoa, Italy; ^3^Department of Psychiatry and Neuropsychology, Maastricht University, Maastricht, Netherlands; ^4^Institute of General Pathology and Pathophysiology, Moscow, Russia; ^5^Laboratory of Psychiatric Neurobiology and Department of Normal Physiology, Institute of Molecular Medicine, Sechenov First Moscow State Medical University, Moscow, Russia; ^6^Kunming Institute of Zoology, Chinese University of Hong Kong Joint Laboratory of Bioresources and Molecular Research in Common Diseases, Chinese Academy of Sciences, Kunming, China

**Keywords:** microglia, neurons, neuroinflammation, transcriptional regulation, microRNA

## Abstract

Twenty years ago, the scientific community exhibited relatively little interest in the study of microglial cells. However, recent technical and conceptual advances in this field have greatly increased interest in the basic biology of these cells within various neurodegenerative diseases, including multiple sclerosis, Alzheimer’s disease, and traumatic brain/spinal cord injuries. The main functions of these cells in the normal central nervous system (CNS) remain poorly understood, despite considerable elucidation of their roles in pathological conditions. Microglia populate the brain before birth and remain in close lifelong contact with CNS-resident cells under the influence of the local microenvironment. Within the CNS parenchyma, microglia actively interact with two main cell types, astrocytes and neurons, which produce many factors that affect microglia phenotypes in the normal CNS and during neuroinflammation. These factors include interleukin (IL)-34, macrophage colony-stimulating factor, transforming growth factor-β, and IL-4, which promote microglial expansion, survival, and differentiation to an anti-inflammatory phenotype in the normal CNS. Under inflammatory conditions, however, astrocytes produce several pro-inflammatory factors that contribute to microglial activation. The interactions of microglia with neurons in the normal and diseased CNS are especially intriguing. Microglia are known to interact actively with neurons by facilitating axonal pruning during development, while neurons provide specific factors that alter microglial phenotypes and functions. This review focuses mainly on the roles of soluble neuronal factors that affect microglial phenotypes and functions and the possible involvement of these factors in the pathology of neurodegenerative diseases.

## Introduction

Microglia are central nervous system (CNS)-resident myeloid cells that originate from common erythro-myeloid progenitors (EMPs), which themselves originate from embryonic stem cells (ESCs) in the yolk. Microglia populate the brain during early fetal development (Kierdorf et al., 2013; Ponomarev et al., 2013). Later, microglia harbor the ability to self-renew locally and remain in the CNS microenvironment throughout life, where they comprise 10–15% of all cells in the brain and spinal cord. In this respect, microglia differ from resident macrophages in other tissues, which originate from hematopoietic stem cells (HSCs) (Li and Barres, 2018), although resident myeloid Langerhans cells in several tissues, such as the skin, represent a mixture of cells derived from ESCs and HSCs (Collin and Milne, 2016). Under pathological conditions such as irradiation or inflammation, HSC-derived myeloid cells may also enter the CNS and acquire the characteristics of ESC-derived microglia, thus leading to a mixed population of HSC- and ESC-derived CNS-resident macrophages similar to that of dermal Langerhans cells (Ponomarev et al., 2013).

Currently, we understand that the phenotypes and functions of microglia are influenced strongly by the local CNS microenvironment. Even in a diseased CNS, microglia in unaffected areas exhibit a normal phenotype and are referred to as homeostatic microglia, whereas microglia proximal to the damaged area exhibit a distinct disease-associated phenotype (Dubbelaar et al., 2018). Under normal conditions, homeostatic microglia are maintained at appropriate densities in various regions of the brain, mostly via local self-renewal (Li and Barres, 2018; Hammond et al., 2019). During disease, microglia in damaged areas often exhibit heterogeneous and mixed-activation phenotypes and may play a beneficial or detrimental role, depending on the area, extent, and nature of neuronal damage and the stage of the disease (Li and Barres, 2018; Szepesi et al., 2018; Hammond et al., 2019). Therefore, it is important to understand how the phenotypes and functions of microglia are determined by specific signals from other CNS-resident cells, such as astrocytes and neurons. This review discusses the nature of such signals and mechanisms of adaptation to the local microenvironment.

## Epigenetic Changes Mediate Adaptation of the Microglia to Tissue-Specific Microenvironments

Modern studies have demonstrated that the adaptation of macrophages to local tissue microenvironments is mediated by epigenetic changes in chromatin (i.e., chromatin remodeling). This process enables the binding of several key transcription factors (TFs) that mediate microglial differentiation (Gosselin et al., 2017; Holtman et al., 2017). Like other tissue-resident macrophages, microglia reside in the CNS under the influence of tissue-derived factors that drive the expression of lineage-specific TFs such as PU.1 and CEBPα (Gosselin et al., 2014). Both CEBPα and PU.1 are induced by macrophage colony-stimulating factor-1 (CSF-1) or its analog, interleukin (IL)-34 (Ponomarev et al., 2013), which are produced by astrocytes and neurons (Frei et al., 1992; Mizuno et al., 2011). These factors drive the survival, renewal, and lineage-specific differentiation of microglia (Li and Barres, 2017). In addition to lineage-specific TFs, tissue-specific TFs complement and modulate the functions of core lineage-specific factors (Gosselin et al., 2014; Holtman et al., 2017), including SMAD family TFs driven by transforming growth factor (TGF) β, which is produced by astrocytes and neurons (Dobolyi et al., 2012). We found that mature microglia very strongly express TGFβ1 mRNA (unpublished observation), indicating that self-TGFβ1 could maintain SMAD2/3 expression in an autocrine manner. Several other factors, including but not limited to V-Maf musculoaponeurotic fibrosarcoma oncogene homolog B (MAFB), interferon response factor (IRF)8, Sal-like (SALL)1, and peroxisome proliferator-activated receptor (PPAR)γ, contribute to the transcriptional control of microglia identity in the normal CNS (Gosselin et al., 2014, 2017; Holtman et al., 2017). Under inflammatory conditions, other inducible TFs, such as nuclear factor (NF)κB, NF-of activated T cells (AT), and signal transducer and activator of transcription (STAT)1/3, contribute further to the phenotypes of activated microglia (Holtman et al., 2017). The cooperative binding of various lineage- and tissue-specific TFs, such as PU.1, CEBPα, IRF8, SMAD2/3, and SALL1, induce the modification of chromatin and generation of primed enhancers for gene expression, which is characterized by monomethylated H3K4 (H3K4me1). In the diseased CNS, inflammatory stimuli [e.g., IL-6, tumor necrosis factor (TNF)] induces the binding of additional factors, such as NFκB, NF-AT, and STAT1/3, and the acetylation of H3K27 (H3K27Ac) (Holtman et al., 2017). Thus, the actions of multiple TFs induce epigenetic changes that lead to the remodeling of chromatin and the formation of a specific mature microglial phenotype. The exact mechanism underlying this highly complex process remains elusive.

## Epigenetic Changes in the Activation and Polarization of Macrophages and Microglia

The phenotypes and functions of immune cells such as microglia and macrophages depend on the type of activation. One example is the existence of pro-inflammatory M1-like macrophages, which are activated by interferon (IFN)γ and/or lipopolysaccharide (LPS), and M2-like macrophages, which are activated by IL-4 and/or IL-13 and associated with the resolution of inflammation and repair of damaged tissues (Sica and Mantovani, 2012; Ley, 2017). The phenotypes of M1 and M2 macrophages are plastic, and the existing model of the activation and polarization spectrum oversimplifies the M1–M2 phenotypic dichotomy, especially *in vivo* (Martinez and Gordon, 2014; Ransohoff, 2016). Nevertheless, *in vitro* studies have clearly delineated a distinct pattern of specific TF expression in M1 macrophages, which is characterized by STAT1, STAT2, IRF4, and NFκB. In contrast, the M2 macrophage phenotype is mediated by STAT6, GATA3, and PPARγ (Lawrence and Natoli, 2011; Cherry et al., 2014; Li et al., 2018). Regarding specific markers, M1 macrophages express nitric oxide (NO) produced by the enzyme NOS2, high levels of major histocompatibility (MHC) class II and CD86 receptors, and the cytokines TNF and IL-6. In contrast, M2 macrophages express arginase (Arg1), extracellular matrix-binding lectin (Ym1/Chi3l3), and the chemokine CCL22, a heparin-binding protein (Lawrence and Natoli, 2011; Krzyszczyk et al., 2018).

Histone methylation and acetylation are associated with both the M1 and M2 states of activated macrophages and microglia (Cheray and Joseph, 2018; Groot and de Pienta, 2018). The methylation of H4R3 has been shown to positively regulate M2 by inducing the expression of the M2-associated TF PPARγ in mouse peritoneal macrophages (PMs) activated with IL-4 (Tikhanovich et al., 2017). H3K4 methylation also positively regulates M2 polarization in human macrophages activated with M-CSF and IL-4 (Kittan et al., 2013). The enzyme JMJD3, known also as H3K27 demethylase, has been shown to be critical to the upregulation of IRF4 and Arg1 in mouse bone marrow-derived macrophages (BMDMs) stimulated with IL-4 (Satoh et al., 2010). The expression of JMJD3 is also upregulated in IL-4-treated mouse microglia, leading to H3K27 demethylation (Tang et al., 2014). Moreover, the acetylation of H3K9 and H3K14 in the *Tnf, Il6*, *Nos2*, and *H2Ab* (MHC class II) promoter regions is a critical inducer of the expression of these M1-associated molecules in LPS-stimulated mouse microglia (Chauhan et al., 2015).

Similar to other tissues, the CNS produces IL-4 via neurons and possibly astrocytes. This cytokine contributes to the expression of M2-like markers and may also induce the expression of the M2-associated TF PPARγ in mature microglia (Veremeyko et al., 2015, 2018b; Zhao et al., 2015). We found that normal microglia express Ym1 in a CNS-derived IL-4-dependent manner (Ponomarev et al., 2007). More recent studies based on single-cell RNA sequencing demonstrated that mouse microglia express *Arg1*, especially during the embryonic and early postnatal stages (Hammond et al., 2019). However, the initial concept of microglial polarization was criticized recently, as microglia were shown to express M1 and M2 markers simultaneously at the single-cell level (Ransohoff, 2016). We found this unsurprising, as we discovered long ago that microglia exhibited dual activation and an M1/M2 mixed phenotype in the context of experimental autoimmune encephalitis (Ponomarev et al., 2007). We believe that criticism of the concept of microglial polarization was mainly due to the misconception that microglia must exist in one of two mutually exclusive states (M1 or M2). More recent studies of macrophages and microglia demonstrated that these activation states are impermanent and somewhat dynamic. We also demonstrated that IL-4-activated macrophages exhibit a high level of plasticity, and that this is due to a high level of Egr2, which enables the cells to react to other activation stimuli (Veremeyko et al., 2015). Interestingly, mouse and human microglia express multiple early response genes, including Egr2, even at a single-cell level, which suggests a high level of cellular plasticity (Masuda et al., 2019). Importantly, therefore, microglia are exposed to a milieu of different cytokines (e.g., IL-4, TNF) during inflammation, and the state of activation represents a dynamic superposition of different activation pathways, rather than the mutual exclusion of the M1 and M2 states.

## Role of Microrna in Microglial Phenotypes and Functions

In addition to histone methylation and acetylation, other epigenetic mechanisms regulate gene expression in microglia. One well-known mechanism involves the suppressive actions of regulatory microRNAs (miRNAs) against gene expression. MiRNAs are short (22–23 nucleotides) non-coding RNAs that bind complementarily to the mRNAs of target genes and either repress the translation or induce the degradation of these mRNAs (Ponomarev et al., 2013). In both cases, translation of the target gene and protein expression are decreased. The roles of miRNAs in microglial functions were reviewed recently (Guo et al., 2019). Moreover, a recent study demonstrated the microglial–neuronal transfer of exosome-associated miRNAs. M1-activated microglia were shown to produce extracellular vesicles containing the microglia-specific miRNA miR-146a-5p, which is not detected in neurons. Upon transfer, this miRNA downregulated the expression of the genes encoding presynaptic synaptotagmin-1 and postsynaptic neuroligin-1 (Prada et al., 2018). In this review, we focus on the roles of two neuronal miRNAs, miR-124 and miR-9, which are transferred from neurons to microglia and/or macrophages. Our screening of microRNAs that are expressed in microglia but not in resident PMs or BMDMs revealed the strong expression of miR-124 in the microglia (Ponomarev et al., 2011). Interestingly, miR-124 is expressed strongly in neurons and is responsible for the differentiation of neuronal progenitor cells (NPCs) into mature neurons (Ponomarev et al., 2013). We determined that miR-124 targeted CEBPα, a critical TF for macrophage and microglial differentiation. CEBPα was expressed in mouse embryonic (until E14) and early postnatal microglia (E15–P14) but not in mature microglia (P60), whereas miR-124 was expressed strongly in mature microglia, but not in embryonic and early postnatal microglia. The transfection of BMDMs with miR-124 decreased the expression of CEBPα and PU.1 and the pro-inflammatory cytokines TNF and IL-6, and increased the expression of the anti-inflammatory factors Arg1, Fizz1, and TGFβ1 (Ponomarev et al., 2011). Indeed, exposure to the pro-inflammatory stimuli TNF and IL-6 increased the expression and DNA binding of CEBPα and PU.1, which in turn increased the induction of downstream gene expression (Crotti et al., 2014). The upregulation of Arg1, Fizz1, and TGFβ1 in macrophages transfected with miR-124 was consistent with the phenotypes of M2-like macrophages activated with IL-4 or IL-13 (Ponomarev et al., 2011). Indeed, we found that IL-4 or IL-13 induced the expression of longer pre-miR-124 transcripts in BMDMs and lung resident macrophages in a STAT6-dependent manner, whereas this process was not observed in mature microglia from a normal CNS (Veremeyko et al., 2013). Therefore, the microglial expression of neuronal miR-124 was upregulated via mechanisms other than the intrinsic transcription of longer miR-124 precursors and subsequent processing and maturation into functional miR-124. Further experiments indicated the horizontal transfer of miR-124 and miR-9 from neurons to microglia (Veremeyko et al., 2018a). Similar to miR-124, miR-9 is expressed strongly in neurons. This miRNA deactivates macrophages and microglia by targeting NFκB, the expression of which is induced in microglia by inflammatory stimuli (Veremeyko et al., 2018a). Therefore, both miR-124 and miR-9 promote an anti-inflammatory phenotype in the normal CNS. These findings also indicate that normal neurons send continuous signals to microglia to maintain the latter cells in a quiescent state.

## Horizontal Transfer of Mirna From Neurons to Microglia

Our understanding of the induction of miR-124 expression in mature microglia led us to hypothesize that this miRNA is transferred from mature neurons to microglia. Although previous reports described the transfer of miRNA between cell types (Barteneva et al., 2013, 2017), most demonstrated the transfer of miRNAs via exosomes or microparticles (Zhang et al., 2015). We investigated in detail the presence of the neuronally abundant microRNAs, miR-124 and miR-9, in the extracellular space. Notably, both miR-124 and miR-9 were secreted actively by electrically active neurons, which was confirmed by using tetrodotoxin (TTX) to block secretion (Veremeyko et al., 2018a). However, both miRNAs were secreted in various forms, including naked single-strand RNA (ssRNA), naked double-strand RNA (dsRNA), and ssRNA or dsRNA complexed with proteins, and within exosomes. Importantly, some proportion of miR-9 was co-localized with exosomes, whereas miR-124 was not associated with exosomes. Rather, miR-124 formed complexes with high-density lipoproteins (HDLs), which are produced in the CNS by astrocytes. These HDL–miR-124 complexes translocated efficiently into the cytoplasm of microglia and macrophages via scavenger receptors. This finding highlights an important mechanism by which functional neurons send continuous signals to the microglia to provide instruction and maintain a resting (non-activated) phenotype in the normal CNS (Veremeyko et al., 2018a). We propose that neuronal electric and synaptic activity decreases, miR-124 and miR-9 secretions are blocked, and the microglial phenotype shifts from resting to activated during neurodegeneration.

## Analogous Regulation of Gene Expression in Microglia and Neurons

We hypothesized that gene expression is regulated similarly, in neurons and microglia, as both cell types are closely associated with each other and exposed to the same microenvironment. Several findings support our hypothesis. First, we observed that microglia express neuronal miRNAs such as miR-124 and miR-9 (Veremeyko et al., 2018a). Second, microglia produce neuronal trophic factors such as brain-derived neurotrophic factor (BDNF) (Parkhurst et al., 2013). Third, microglia express Egr1, c-Jun, and c-Fos, which are products of neuronal early response genes (Holtman et al., 2017). Fourth, microglia express Sall1, which, together with Egr1, is associated with neuronal maturation and synaptic pruning (Buttgereit et al., 2016). Egr1, c-Jun, and c-Fos are hallmarks of neuronal synaptic activity (Dukhinova et al., 2018), while Egr2 is involved in macrophage activation, polarization, and plasticity (Veremeyko et al., 2015). However, c-Fos forms complexes with c-Jun to form activator protein-1 (AP-1), a very potent TF associated with macrophage activation (Hop et al., 2018). AP-1 induces the expression of pro-inflammatory genes in macrophages and microglia (Matcovitch-Natan et al., 2016; Holtman et al., 2017). Alone, c-Fos suppresses the expression of M1-associated genes such as *Nos2*, *Tnf*, and *Il6* (Okada, 2003; Ray et al., 2006). CEBPβ is another interesting TF that is regulated in macrophages by Egr1–3 and the cAMP pathway (Veremeyko et al., 2015, 2018b). CEBPβ is important for cortical neurogenesis and may thus induce the expression of both myeloid and neuronal genes (Ménard et al., 2002). Sall1 also regulates cortical neurogenesis (Harrison et al., 2012) and serves as a unique marker of microglia. The miRNA miR-124 inactivates the transcriptional repressor REST, which in turn inhibits the expression of neuronal genes in non-neuronal cell types (Visvanathan et al., 2007). Mir-124 is expressed in microglia and M2 macrophages. Sall1-deficient or miR-124 inhibitor-treated microglia lose their processes that are typical for adult microglia in normal CNS (ramified form) and take a more activated amoeboid form, indicating that the resting microglial phenotype might be maintained by neuronal genes (Ponomarev et al., 2011; Koso et al., 2016, 2018). In other words, neurons and mature microglia share similar expression patterns for several genes. We believe that these neuronal genes may contribute to the unique phenotypes of microglia in the CNS. However, the induction of neuronal gene expression in microglia remains unclear and must be addressed. We propose that neuronal gene expression in microglia is associated with similar epigenetic changes that occur in both cell types during development. During neural lineage commitment, H3K27 in the promoter regions of many neural lineage genes is demethylated by the enzyme JMJD3 (Choi et al., 2015), similar to the effects of IL-4 on mouse BMDMs. Simultaneously, H3K27 is methylated in non-neuronal cells (including non-activated peripheral macrophages) (Satoh et al., 2010). These findings indicate similar epigenetic changes in microglia, M2 macrophages, and neuronal cells.

**FIGURE 1:**
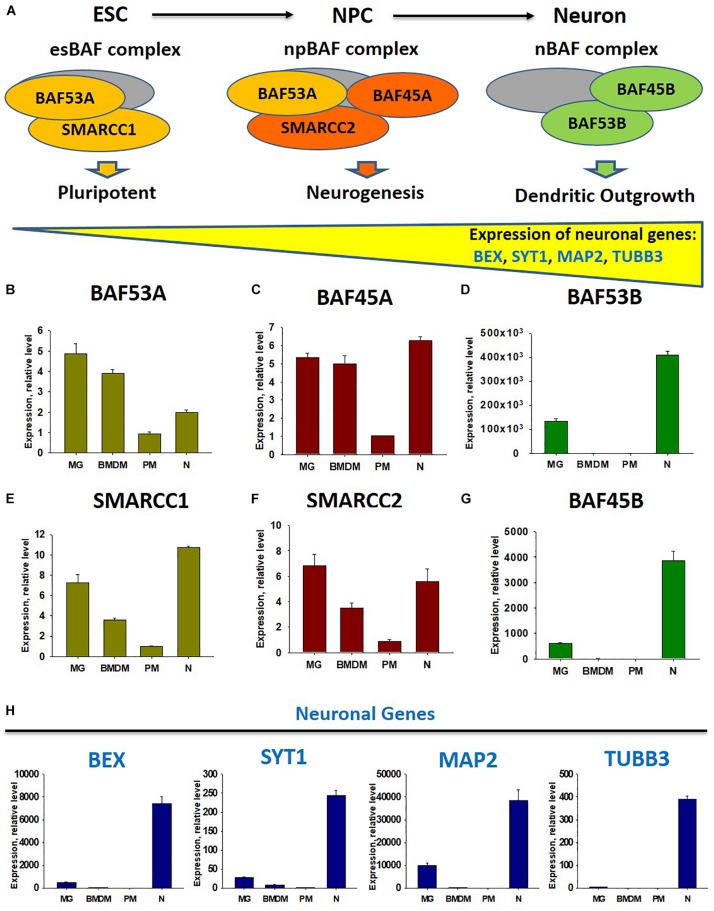
Comparison of expression of factors of chromatin remodeling BAF complexes at the stage of embryonic stems cells (esBAF), neuronal progenitor cells (npBAF), and neurons (nBAF) as well as analysis of expression neuronal genes in microglia, bone marrow-derived macrophages, peritoneal macrophages, and cultured cortical neurons. **(A)** Schematic diagram of Brm/Brg-associated factor (BAF) chromatin remodeling complex for three stages of neuronal differentiation from embryonic stem cells (ESC) to neuronal progenitor cells (NPC) and to mature neurons (Neuron) (Adapted from “Epigenetic regulation of neural differentiation from embryonic stem cells” by Atsushi Shimomura and Eri Hashino with permission. 2013 Shimomura A., Hashino E. Published in *Trends in Cell Signaling Pathways in Neuronal Fate Decision* under CC BY 3.0 license. Available from: 10.5772/53650). **(B–G)** Expression of factors of BAF complex *BAF53A* **(B)**, *BAF45A* **(C)**, *BAF53B* **(D)**, *SMARCC1* **(E)**, *SMARCC2* **(F)**, and *BAF45B* **(G)** in microglia (MG), bone marrow-derived macrophages (BMDM), peritoneal macrophages (PM), and cultured cortical neurons (N). **(H)** Expression of neuronal genes *BEX*, *SYT1*, *MAP2*, and *TUBB3 i*n microglia (MG), bone marrow-derived macrophages (BMDF), peritoneal macrophages (PM), and cultured cortical neurons (N).

Besides the demethylation of H3K27 in M2 macrophages, miR-124 is upregulated by the IL-4 and cAMP pathways in mouse BMDM (Veremeyko et al., 2018b), while microglia receive horizontal transfers of miR-124 and miR-9 from neurons (Veremeyko et al., 2018a). Therefore, we propose that neuronal miR-124 and miR-9 inhibit REST in microglia, leading to the expression of several neuronal genes, similar to the consequences of miR-124 and miR-9 overexpression in fibroblasts (Lee et al., 2018). As the deactivation of REST and upregulation of miR-124 were shown to be associated with the modification of the neuronal BAF chromatin remodeling complex during neuronal lineage commitment (Choi et al., 2015), we hypothesized that BAF would also be modified in the microglia, compared with non-activated BMDM. To test this hypothesis, we investigated the neuronal BAF chromatin remodeling complex (Choi et al., 2015), which regulates the expression of neuronal genes during differentiation from embryonic stem cells (ESCs) to neuronal progenitor cells (NPCs) from NPCs to mature neurons (Shimomura and Hashino, 2013), in neurons, peritoneal macrophages (PMs), BMDMs, and microglia (Figure 1A). In the ESC stage, the esBAF complex exists, and the co-factors *BAF53A* and *SMARCC1* are expressed predominantly. In the NPC stage, the npBAF complex retains *BAF53A* but includes two new factors, *BAF45A* and *SMARCC2.* Finally, in mature neurons, the nBAF complex comprises *BAF45B* and *BAF53B*. The expression of the neuronal genes *BEX*, *SYT1*, *MAP2*, and *TUBB3* increased gradually during the ESC→ *NPC*→neuron transition path (Figure 1A). We similarly compared the expression levels of transcripts encoding factors in the esBAF, npBAF, and nBAF complexes in mature microglia, BMDMs, PMs, and cultured mature cortical neurons, as described in our earlier studies (Veremeyko et al., 2015, 2018a; Dukhinova et al., 2018, 2019). We observed a similar expression of *BAF53A* and *SMARCC1*, which encode esBAF components, between microglia and neurons and between microglia and BMDMs. However, these genes were expressed at significantly higher levels in microglia than in PMs (Figures 1B,E). We observed a similar pattern in the expression of *BAF45A* and *SMARCC2*, which encode npBAF components. These genes were expressed similarly in microglia and neurons, at similar or slightly higher levels in microglia relative to BMDMs, and at significantly higher levels in microglia than in PMs (Figures 1C,F). However, *BAF53B* and *BAF45B*, which encode nBAF components, were only expressed at detectable levels in neurons and microglia, but not BMDMs and PMs (Figures 1D,G). The neuronal genes *BEX*, *SYT1*, and *MAP2* were expressed in neurons and microglia, but not in BMDMs and PMs (Figure 1H). *TUBB3*, the most abundant neuronal gene, was not expressed in microglia, indicating that the previous results could not be attributed to the contamination of microglia preparations with neuronal cells (Figure 1H, *TUBB3*). Therefore, in contrast with other macrophages, microglia express factors associated with the nBAF complex, as well as several neuronal genes. Although the roles of neuronal gene products such as MAP2 in the microglial phenotype remain unclear, we propose that these factors contribute to the unique phenotype of mature microglia in the normal CNS.

## Roles of Neuronal Soluble Factors in the Maintenance of the Microglial Phenotype

**FIGURE 2:**
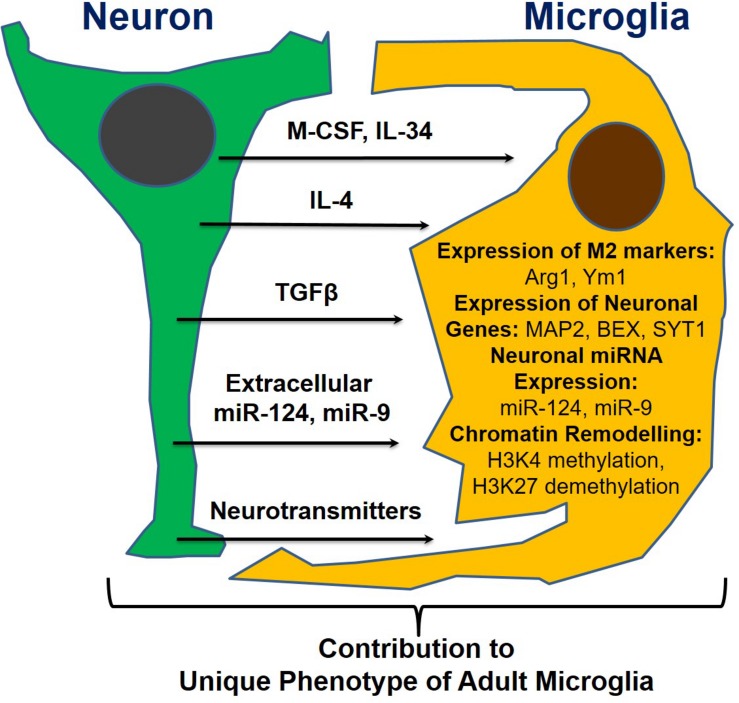
Neuronal soluble factors that determine the ultimate phenotype of microglia in the normal or diseased CNS.

Following our discovery of the horizontal transfer of neuronal miR-9 and miR-124 from electrically active neurons to microglia, we hypothesized that the microglial phenotype is influenced strongly by soluble neuronal factors (Table 1 and Figure 2). Accordingly, these soluble neuronal factors exhibit great potential for future therapeutic interventions targeting neurodegenerative diseases. Both astrocytes and neurons can produce IL-4, TGFβ1, and IL-34, which greatly affect the microglial phenotype, as discussed previously (Table 1). Neuronal IL-4 might further contribute to H3K27 demethylation in microglia (Cheray and Joseph, 2018). Neurons also produce the chemokines CCL2, CCL12, and CX3CL1 (both membrane-bound and soluble forms), which can induce microglial migration toward neurons (CCL2 and CCL12) and microglial deactivation (CX3CL1) (Haas et al., 2019). Neuronal growth factor (NGF) and BDNF also contribute to the anti-inflammatory microglial phenotype (Table 1; Lai et al., 2018; Fodelianaki et al., 2019). Neurotransmitters are other important molecules released by electrically active neurons that can directly affect microglia. Common neurotransmitters, such as serotonin, dopamine, and GABA, deactivate microglia, while glutamate activates these cells (Table 1; Liu et al., 2016). ATP is released by activated and damaged neurons and contributes to microglial activation and inflammation (Davalos et al., 2005). The neuronal microRNAs miR-124 and miR-9 are transferred to the microglial cytoplasm, where they downregulate the expression of CEBPα and NFκB and deactivate the cells (Table 1; Veremeyko et al., 2018a). Finally, the complement components C1q and C3 mediate neuronal axonal pruning by microglia via complement receptors (Table 1). Interestingly, C1q expression is induced in neurons via a TGFβ1-mediated mechanism, while C3 is present in neuronal synapses (Luchena et al., 2018). In summary, many factors, including pro- and anti-inflammatory stimuli, affect communication between neurons and microglia. The balance of these stimuli determines the ultimate phenotype of microglia in the normal or diseased CNS (Figure 2).

**TABLE 1:** Neuron-derived soluble factors that affect the activation state of microglia in normal CNS and during neuroinflammation and neurodegeneration.

**Class of mediators**	**Neuron-derived mediators**	**Changes during neuroinflammation/neurodegeneration**	**Effect on microglia**
Cytokines	IL-4	Decreased	Promote anti-inflammatory phenotype and expression of: Ym1, Fizz1, TGFβ1 (Veremeyko et al., 2015, 2018b; Zhao et al., 2015; Cheray and Joseph, 2018)
	TGFβ1	Decreased	Promote homeostatic phenotype of microglia in normal adult CNS (Dobolyi et al., 2012)
	IL-34	Increased	Homeostatic maintenance, proliferation, and survival of microglia (Frei et al., 1992; Mizuno et al., 2011; Ponomarev et al., 2013)
Chemokines	CCL2	Increased	Microglia migration, activation, and proliferation (Haas et al., 2019)
	CXCL12	Increased	Microglia migration and activation (Haas et al., 2019)
	CX3CL1 (soluble form)	Decreased	Deactivate microglia (Haas et al., 2019)
Growth Factors	NGF	Increased	Promote anti-inflammatory phenotype of microglia (Fodelianaki et al., 2019)
	BDNF	Increased	Suppress release of NO in microglia. Promote anti-inflammatory phenotype (Lai et al., 2018)
Neurotransmitters	Serotonin (5HT)	Decreased during TBI-induced neuroinflammation	Decreased IFNγ-induced activation of macrophages and possibly microglia (Liu et al., 2016)
	Dopamine	Decreased	Possibly suppress microglia activation via DR2 (Liu et al., 2016)
	Glutamate	Decreased	Promote microglia activation and expression of TNF and NO (Liu et al., 2016)
	GABA	Decreased	Inhibit microglia activation and release LPS-induced TNF and IL-6 (Liu et al., 2016)
Low-molecular-weight mediators	ATP	Increased	Activate microglia (Davalos et al., 2005)
microRNAs	miR-124 miR-9	Decreased Decreased	Deactivate microglia (Veremeyko et al., 2018a)
Complement	C3	Increased	Mediate synaptic pruning by microglia (Luchena et al., 2018)
	C1q	Increased	

## Concluding Remarks

Microglia represent a unique population of resident myeloid cells in the CNS. The phenotypes of these cells are influenced strongly by the local microenvironment and determined by the specific regulation of gene expression at the epigenetic level. Currently, neuronal cells are thought to actively influence the phenotypes of microglia. In the normal CNS, electrically and synaptically active neurons maintain microglia in a deactivated state, whereas changes in the balance of inhibitory vs. activating stimuli in the contexts of neuroinflammatory and neurodegenerative diseases cause immediate changes in the microglial phenotype and induce activation. A further understanding of the interactions of neurons and microglia in the normal CNS would ultimately lead to the discovery of new pathways. This information would suggest potential mechanisms by which homeostasis could be restored in pathological settings, as well as potential treatment options for neurodegenerative diseases such as multiple sclerosis, Alzheimer’s disease, and Parkinson’s disease.

## Author Contributions

TV and EP conceived the review and study. TV, AY, and MD performed the experiments. TV, TS, and EP analyzed the data. TS and EP prepared the manuscript.

## Conflict of Interest

The authors declare that the research was conducted in the absence of any commercial or financial relationships that could be construed as constituting a potential conflict of interest.
